# Thromboelastometry for Assessing Risks of Free Flap Thrombosis in Patients Undergoing Microvascular Surgery

**DOI:** 10.3389/fmed.2020.00289

**Published:** 2020-06-23

**Authors:** Indulis Vanags, Jevgenijs Stepanovs, Agnese Ozolina, Maksims Mukans, Lars J. Bjertnaes, Biruta Mamaja

**Affiliations:** ^1^Department of Anaesthesiology and Reanimatology, Riga Stradinš University, Riga, Latvia; ^2^Department of Anaesthesiology and Intensive Care Unit, Pauls Stradins Clinical University Hospital, Riga, Latvia; ^3^Department of Anaesthesiology, Riga East Clinical University Hospital, Riga, Latvia; ^4^Statistical Unit, Riga Stradinš University, Riga, Latvia; ^5^Anesthesia and Critical Care Research Group, Department of Clinical Medicine, Faculty of Health Sciences, University of Tromsø, Tromsø, Norway

**Keywords:** free flap thrombosis, hypercoagulability, microsurgery, rotational thromboelastometry (RTEM), risk

## Abstract

**Introduction:** Coagulation assessment is often missing in microvascular surgery. We aimed at evaluating the predictive value of thromboelastometry for free flap thrombosis in microvascular surgery patients.

**Materials and Methods:** We enrolled 103 adult patients with traumatic injuries scheduled for microvascular free flap surgery into a prospective observational study. Thirty-six patients with recent trauma underwent surgery within 30 days (ES group), and were compared with 67 trauma patients who underwent surgery later than 30 days (late surgery, LS group) after the injury. Rotational thromboelastometry (RTE) was performed before surgery. Functional fibrinogen to platelet ratio (FPR) ≥ 42 was selected as the main hypercoagulability index. Free flap thrombosis was set as primary outcome. Thrombotic risk factors and duration of surgery related to free flap thrombosis were secondary outcomes. Statistical significance *p* < 0.05; not significant NS.

**Results:** Six patients (16.7%) in the ES group and 10 (14.9%) in the LS group had free flap thrombosis (NS). In the entire cohort, free flap thrombosis rate increased in the presence of thrombogenic comorbidities (OR 4.059, CI 1.33–12.37; *p* = 0.014) and prolonged surgery times (OR 1.007, CI 1 – 1.012; *p* = 0.05). Although hypercoagulability occurred more frequently in the ES group (44.4%) than in the LS group (11.9%; *p* < 0.001), it was not associated with higher free flap thrombosis rate. In ES group patients with surgery times > 240 min, the risk of free flap thrombosis increased (OR 3.5, CI 1.16-10.6; *p* = 0.026) with 93.3% sensitivity and 86.7% specificity (AUC 0.85; *p* = 0.007). In contrast, in LS patients hypercoagulability increased the odds of free flap thrombosis (OR 8.83, CI 1.74–44.76; *p* = 0.009). Moreover, a positive correlation was found between FPR ≥ 42 and free flap thrombosis rate (*r* = 0.362; *p* = 0.003). In the LS group, the presence of thrombogenic comorbidities correlated with free flap thrombosis rate (OR 7, CI 1.591–30.8; *p* = 0.01).

**Conclusions:** In LS patients with thrombogenic comorbidities, thromboelastometry supports the detection of hypercoagulability and predicts free flap thrombosis risk. In ES patients, postoperative hypercoagulability did not predict free flap thrombosis. Prolonged surgery time should be considered as a risk factor.

## Introduction

Microvascular free flap surgery allows closure of various tissue defects in reconstructive microsurgery. Since the introduction of free tissue transfer, advancements in microsurgical techniques have increased flap survival rates (1–3). However, despite technical progress, free flap thrombosis still remains a matter of concern (4, 5) because it is associated with higher morbidity, increased costs and, longer hospital stay. Ultimately, it might lead to free flap failure (6). Although, microvascular free flap thrombosis most often occurs secondary to technical mishaps and/or longer duration of surgery (7), a number of other preoperatively identifiable factors have a potential impact on anastomotic thrombosis rate and outcome of tissue transfer (8–10). Patient - related factors, such as hereditary or acquired hypercoagulability (11–14), age (8), diabetes mellitus (15, 16), poor nutritional status (17), cardiovascular diseases (6, 18) or autoimmune disorders (11) were all demonstrated to affect negatively the outcomes of tissue transfer surgery.

In trauma patients, a time-dependent hemostatic response initially increases the risk of bleeding due to hemodilution or consumption coagulopathy in combination with a rise in fibrinolytic activity (19). Subsequently, a period of increased pro-coagulant activity follows (20). Diagnosing a condition of posttraumatic hypercoagulability is complex and no standardized tests unequivocally give the answer. Thrombin generation tests, or assessment of plasma levels of natural anticoagulants, as protein C and S, are seldom available for clinical use and not validated for detection of hypercoagulability. Routine coagulation tests like activated partial thromboplastin time (APTT) and prothrombin time (PT) are widely used to manage anticoagulation therapy, but not for assessing the risk of excessive bleeding or thrombosis. Several investigations have shown that fibrinogen plasma levels of <1.5 g/L correlate highly with increased risks of bleeding (21–23). In contrast, there is no clear association between free flap thrombosis rate and higher fibrinogen plasma levels. Besides that, a history of recent traumatic injury in combination with posttraumatic coagulation changes might increase microvascular thrombosis rate. Cho and co-authors reported a 4-fold increased chance of lower extremity free flap thrombosis and a doubling of the risks of postoperative thrombosis in patients of acute trauma with preoperative thrombocytosis (14).

In recent years, rotational thromboelastometry (RTE) has become an extensively used method of monitoring coagulant activity in major bleeding after trauma (24–26), cardiac surgery (27, 28), and other conditions that might lead to great blood losses (29). The method integrates real-time information on all aspects of coagulation, including the presence of hypercoagulability (30, 31). However, there are few reports on the use of RTE for predicting the risks of free flap thrombotic complications in patients undergoing microvascular surgery, and the method is still controversial (32–34). We hypothesize that hypercoagulability timely detected by RTE, can indicate a higher risk of free flap thrombosis. Therefore, the aim of this study was to assess the role of RTE as a means of early identification of hypercoagulability in trauma patients prone to develop free flap thrombosis. Hypercoagulability related to free flap thrombosis until hospital discharge, was considered as a primary outcome, and thrombotic risk factors and duration of surgery were evaluated as secondary outcomes.

## Materials and Methods

### Population

We enrolled 103 subsequent adult patients with traumatic injuries scheduled to undergo microvascular free flap surgery at the Latvian Center of Reconstructive and Microsurgery, Riga, Latvia into a prospective observational study. We compared two groups of patients according to the time that had elapsed from the traumatic event until the day of surgery. Thirty-six patients, who were operated within a 30-day period after trauma, during the primary hospital stay, were allocated to an early surgery group (ES group), and the remaining 67 patients were referred to a late surgery group (LS group) that was operated later than 30 days after the injurious trauma due to late complications, such as infections or scars. In those cases, tissue defects were closed secondary in the late posttraumatic period.

The Ethics Committee of Riga Stradinš University approved the study protocol (Nr.1/26-11-16) and the informed consent form. All adult patients with traumatic injuries who underwent microvascular free flap surgery during the study period were asked to participate in the study. We excluded patients below 18 years of age and patients with ongoing oncological treatment or history of confirmed thrombophilia or pregnancy. Surgery was performed as a consequence of acute or chronic traumatic injuries. All patients gave their prior written informed consent.

### Perioperative Management

General anesthesia (GA) was provided for all enrolled patients according to the guidelines for free flap transfer surgery. After induction with fentanyl (Fentanyl-Kalceks® 0.05 mg/ml, A/S Kalceks, Latvia) 1.5–2 μg/kg, and propofol (Propofol® 10 mg/ml, Fresenius Kabi AG, Germany) 1–2 mg/kg intravenously (iv), GA was maintained with sevoflurane (Sevorane®, AbbVie S.r.l., Italy) 0.8–1.2 MAC, and fentanyl 1–1.5 μg/kg/h. We administered cisatracurium (Nimbex 2 mg/ml, Aspen Pharma Ltd, Ireland) 0.15 mg/kg iv for tracheal intubation, followed by a continuous infusion of 1–2 μg/kg/min for muscle relaxation. Crystalloid infusion (RiLac, B. Braun Melsungen AG, Germany) of 3.5 to 6.0 ml/kg iv per hour during the 24-h perioperative period was standardized and controlled for adequacy guided by monitoring of a urine output of 1–2 ml/kg/h. To avoid hypothermia, we measured central (esophageal probe) and peripheral (axillary probe) temperatures (°C) of the patients, maintaining core temperatures above 35°C and a difference between central and peripheral measurements (Δt) of <2°C. We assessed patients' volume status by evaluating urine output and Δt combined, in addition to hemodynamic parameters. The decision to insert a line for central venous pressure monitoring was guided by the patient's cardiovascular status, anticipated blood loss and need of vasoactive substances.

We performed peripheral blocks under ultrasound guidance when indicated. We applied brachial plexus block with axillary approach for upper extremity surgery, femoral nerve and popliteal blocks for lower extremity postoperative analgesia. Paracetamol (Paracetamol, B. Braun Melsungen AG, Germany) 1.0 g four times daily in combination with non-steroidal anti-inflammatory drugs two times daily were used for postoperative multimodal analgesia. For those without a peripheral nerve block, we administered a continuous intravenous infusion of fentanyl 0.5–1 mcg/kg/h.

Postoperatively, tightly followed hemodynamic monitoring, fluid management and oxygen supply was provided in the post-anesthesia care unit. According to hospital guidelines, postoperative thrombotic prophylaxis was provided with enoxaparin 40 mg (CLEXANE®, Sanofi-Aventis S.A. Spain) once daily from the 1st postoperative day for all included patients. Hemoglobin and hematocrit levels were optimized (Hb > 10 g/dl, Hct > 30%) in anemic patients with allogenic hemotransfusions before surgery. During and after surgery, patients with clinical symptoms of excessive blood loss or those with Hb <7 g/dl received blood product transfusions.

During the study, the same team of specialists trained in microsurgery performed all microvascular flap surgeries. The surgeon monitored the microvascular flap for 3–5 days postoperatively. Clinical assessment of flap color and temperature, tissue turgor and capillary refill were evaluated as flap perfusion criteria. In all cases of free flap thrombosis, urgent surgical re-exploration was performed in order to salvage the transferred free flap. The venue of thrombosis (e.g., arterial, venous or both) was assured by direct visualization during revision of the anastomosis.

### Study Protocol

Preoperatively, a blood sample was drawn for RTE (ROTEM®, Tem Innovations GmbH, Munich, Germany) assessment of the coagulation status. For each RTE test, we used 300 μl of citrated blood with 20 μl of 0.2 mol/L CaCl_2_ for recalcification. As activators, we added 20 μl of recombinant tissue factor for EXTEM test, 20 μl of partial thromboplastin phospholipid from rabbit brain for INTEM test, and 20 μl cytochalasin D for FIBTEM test. For all three test assays, we recorded the following parameters of RTE: clotting time (CT, period from start of clot formation to the first visible clotting; normal range for EXTEM 38–79 s, normal range for INTEM 100–240 s); clot formation time [CFT, time to the clot reached a diameter of 20 millimeters (mm); normal range for EXTEM 34–159 s, normal range for INTEM 30–110 s], MCF-maximum clot firmness (the maximum clot diameter in mm; normal ranges for EXTEM and INTEM 50–72 mm, normal range for FIBTEM 9–25 mm) (35). Hypercoagulability was defined as functional fibrinogen to platelet ratio (FPR) higher or equal to 42, as previously described by Parker et al. (32). To calculate FPR maximum, we divided clot firmness of FIBTEM (MCF_FIBTEM_) by MCF_INTEM_, as expressed by a numerical ratio.

In parallel, we performed standard coagulation measurements. Prothrombin index (PI, normal range 70–130%), activated partial thromboplastin time (APTT, normal range 28–40 s) and fibrinogen plasma levels (normal range 2–4 g/L) were analyzed in citrated plasma using the STA-R COMPACT (Diagnostika Stago, France). Platelet count (normal range 150–400 × 10^9^/L) was determined by using Sysmex XE-5000 (Sysmex Kobe, Japan).

In search of thrombogenic risk factors, the patients were interviewed in order to register co-morbidities, particularly emphasizing histories of previous thrombotic events. In parallel, we noted demographic data, localization of soft tissue defect and the duration of surgery. We defined trauma as recent if the free flap had been transferred within 30-days after the injury.

### Statistical Analysis

Data were analyzed with SPSS Statistics (Chicago, IL, Version 20). We assessed the data distribution with Shapiro-Wilk test because the sample size was small. Continuous variables were presented as mean ± standard deviation (SD) or median with interquartile range (IQR), as appropriate, and categorical variables as percentages (%). Quantitative (continuous) data were non-normally distributed (except for age of patients). Therefore, we used non-parametric Kruskal-Wallis H test to compare differences between quantitative data. Cross tabulation was used to compare free flap thrombosis (yes/no), time to operation (ES, LS) and demographics, clinical data and RTE variables. To assess statistical differences between the abovementioned data, we used Pearson Chi Square test and if small sample size (<5 cases) Fisher's exact test. Correlations between free flap thrombosis factors were performed with non-parametric Spearman rho test. ROC curve analysis was performed to state the diagnostic value of FPR for free flap thrombosis with threshold level of ≥ 42. We employed ROC curve analysis to find the thresholds for sensitivity and specificity of FPR, and duration of surgery and furthermore, to find the threshold of operation time at which the risk of thrombosis is high. Linear regression was performed to find adjusted predicted values of thrombosis for the length of operation. We also performed binary regression analysis in the entire population and in subgroups of early (ES group) and late surgery (LS group) patients to find significant free flap thrombosis factors. Statistical significance was reached at alpha level <0.05.

## Results

### Clinical Course

Table 1 displays demographic and clinical variables of 90 men and 13 women fulfilling the inclusion criteria and all requirements of the protocol, including thrombogenic risk factors, localization of tissue defects and durations of surgery.

**Table 1:** Comparison of demographic data, thrombogenic risk factors, trauma localization, and duration of surgery between patients of the early surgery (ES) group and the late surgery (LS group).

**Demographic data**	**ES group, *n* = 36**	**LS group, *n* = 67**	***p*-value**
Age, years, mean	37.6 (SD = 13)	42.4 (SD = 12)	NS
Gender, female, n (%)	4 (11.1)	9 (13.4)	NS
**Thrombogenic risk factors, n (%)**			
Thrombogenic comorbidities^*^	4 (11.1)	21 (31.8)	0.029
Chronic osteomyelitis	0	22 (32.8)	<0.001
Smoking	16 (44.4)	23 (34.3)	NS
Thrombocytosis	10 (27.8)	11 (16.4)	NS
Hyperfibrinogenemia, > 4 g/L	23 (63.9)	23 (34.3)	<0.001
**Trauma localization, n (%)**			
Lower extremity	30 (83.3)	40 (59.7)	0.014
Upper extremity	6 (16.7)	21 (31.3)	NS
Lumbar region	0	2 (3)	NS
Head	0	4 (6)	NS
Duration of surgery, min, median (IQR)	215 (243–190)	225 (300–190)	NS

*Data are presented as mean ± SD or number (n) or median and interquartile range (IQR). n, number of patients; %, per cent; NS, non-significant difference; SD, standard deviation*.

* *Thrombotic comorbidities: history of previous thrombosis, ischemic disease, hypertension, obesity, neuroparesis, diabetes*.

Demographic data did not differ significantly (NS) between the groups (Table 1). As expected, thrombogenic risk factors like chronic osteomyelitis (*p* < 0.001) and thrombogenic comorbidities (*p* = 0.029) appeared significantly more often in the LS group, whereas posttraumatic hyperfibrinogenemia with fibrinogen plasma levels > 4 g/L were registered more frequently in the ES group (*p* < 0.001). There were no significant differences in smoking history, thrombocytosis or surgery time between the groups. Interestingly, the ES group patients underwent mainly lower extremity reconstruction (83.3%). The most frequent locations of myocutaneus free flap surgeries were on the scapula and in the parascapular area (21.3%), as well as in the anterolateral area of the thigh (12.6%) and the latissimus dorsi muscle area (13.5%). Fibula transposition was the most frequent bone tissue type (9.7%).

### Preoperative Coagulation, Blood Variables, and RTE Parameters

As shown in Table 2, we observed no differences between the two groups in preoperative APTT, PI and platelet count. In contrast, when comparing ES and LS group patients, the former presented significantly more often with a lower Hb level (*p* < 0.001), and higher fibrinogen plasma concentration (*p* = 0.001), that is characteristic for the acute posttraumatic period.

**Table 2:** Comparison of hematological variables, conventional coagulation tests, and rotational thromboelastometry (RTE) variables between early surgery (ES group) and late surgery (LS group) patients.

**Parameters**	**ES group, *n* = 36**	**LS group, *n* = 67**	***p* value**
**Hematological variables, mean** **±** **SD**			
Hb, g/dL	11.1 ± 1.6	13.6 ± 2.4	<0.001
PLT, 10^9^/L	358 (435–208)	281 (337–239)	NS
**Conventional tests, mean, (max-min)**			
Fibrinogen, g/L	4.41 (5.45–3.7)	3.74 (4.62–2.95)	0.003
APTT, sec	33 (35–29.9)	32.9 (35.3–29.4)	NS
PI, %	96 (106–75)	101 (111–87)	NS
**RTE parameters, mean (max-min)**			
CT _EXTEM_	59.5 (68–54)	62 (72–56)	NS
CFT _EXTEM_	48.5 (68–40)	71 (87–52)	0.001
MCF _EXTEM_	73.5 (77–68)	66 (72–63)	0.001
CT _INTEM_	164 (193–137)	185 (233–149)	0.013
CFT_INTEM_	47.5 (61–40)	69 (89–54)	<0.001
MCF _INTEM_	70 (75–66)	65 (69–61)	0.001
CT _FIBTEM_	55.5 (65–48)	57 (64–52)	NS
CFT_FIBTEM_	76 (160–52)	139 (503–36)	0.002
MCF _FIBTEM_	26.5 (34–21)	19 (25–15)	<0.001

*Data are presented as mean ± SD or mean (max–min). APTT, activated partial thromboplastin time; sec, seconds; CT, clotting time; CFT, clot formation time; Hb, hemoglobin concentration; MCF maximum clot firmness; PLT, platelet count; PI, prothrombin index; NS, non-significant difference*.

Assessing preoperative RTE parameters, significant shortening of clot formation time (CFT) and increase in maximum clot firmness (MCF) were detected in the ES group. In contrast, we found no differences between the groups with regard to plasma coagulation, as evidenced by clotting time (except CT _INTEM_, *p* = 0.013).

### Association of Hypercoagulability and Free Flap Thrombosis

Free flap thrombosis developed within the first 24 h after surgery in 16 patients (15.5 %) without significant intergroup difference (Table 3).

**Table 3:** Incidence of free flap thrombosis and hypercoagulability in early surgery (ES group) and late surgery (LS group) patients.

**Primary outcome**	**Totally,*n* = 103**	**ES group, *n* = 36**	**LS group, *n* = 67**	***p* value**
Free flap thrombosis, n (%)	16 (15.5%)	6 (16.7%)	10 (14.9%)	NS
Hypercoagulability, FPR ≥ 42, n (%)	24 (23%)	16 (44.4)	8 (11.9)	<0.001

*Data are presented as categorical variable with number (n) and percentage (%); NS, non-significant difference*.

Hypercoagulability detected by RTE was seen in totally 24 patients, and more often in the ES group (*p* < 0.001), as demonstrated in Table 3. Hypercoagulability presented as a significant risk factor for free flap thrombosis (OR 8.83, CI 1.74–44.76; *p* = 0.009) in the LS group patients, as depicted in Table 4. Additionally, in the same group we found positive correlations between free flap thrombosis rate and FPR ≥ 42 (*r* = 0.362; *p* = 0.003), MCF _EXTEM_ (*r* = 0.257; *p* = 0.036), and MCF _FIBTEM_ (*r* = 0.257; *p* = 0.036).

**Table 4:** Thrombogenic risk factors and duration of surgery in association with free flap thrombosis in early surgery (ES group) and late surgery (LS group) patients.

	**ES group**, ***n*** **=** **36**			**LS group**, ***n*** **=** **67**		
**Variables**	**Odds ratio**	**Confidence interval (95%)**	***p* value**	**Odds ratio**	**Confidence interval (95%)**	***p* value**
**Thrombogenic risk factors**						
Smoking	1.308	(0.226–7.568)	NS	0.793	(0.185–3.406)	NS
Age	0.94	(0.867–1.019)	NS	1.014	(0.958–1.073)	NS
Thrombogenic comorbidities^*^	1.8	(0.154–20.98)	NS	7	(1.591–30.8)	0.010
Hypercoagulability (FPR >42)	0.571	(0.091–3.608)	NS	8.83	(1.743–44.76)	0.009
**Duration of surgery**						
Duration of surgery, min	1.007	(1.000–1.012)	0.05	1.007	(1.000–1.012)	0.05

* *Thrombogenic comorbidities: history of previous thrombosis, ischemic disease, hypertension, obesity, neuroparesis, diabetes*.

Although 44.4% of the patients in the ES group had hypercoagulability (FPR ≥ 42), it did not affect the rate of free flap thrombosis.

### Association of Thrombogenic Risk Factors and Duration of Surgery in Free Flap Thrombosis

By assessing the possible influence of thrombogenic risk factors and duration of surgery on free flap thrombosis rate in all the enrolled patients, we found that those with previous thrombogenic comorbidities had a 4-fold increased odds of free flap thrombosis (OR = 4.059; *p* = 0.014). The latter patients were more often presented in LS group. Additionally, multifactorial analysis showed that combination of hypercoagulability in RTE and time to surgery increases risk for free flap thrombosis (OR = 1.01; *p* = 0.016) in the whole population.

### Late Surgery Group

In addition to hypercoagulability (FPR ≥ 42), as a marked risk factor for free flap thrombosis, a history of thrombogenic co-morbidities demonstrated an influence on flap thrombosis rate (OR 7, CI 1.591–30.8); *p* = 0.01. A positive correlation was detected between hypercoagulability and the presence of thrombogenic comorbidities r = 0.277; *p* = 0.024.

### Early Surgery Group

**Figure 1:**
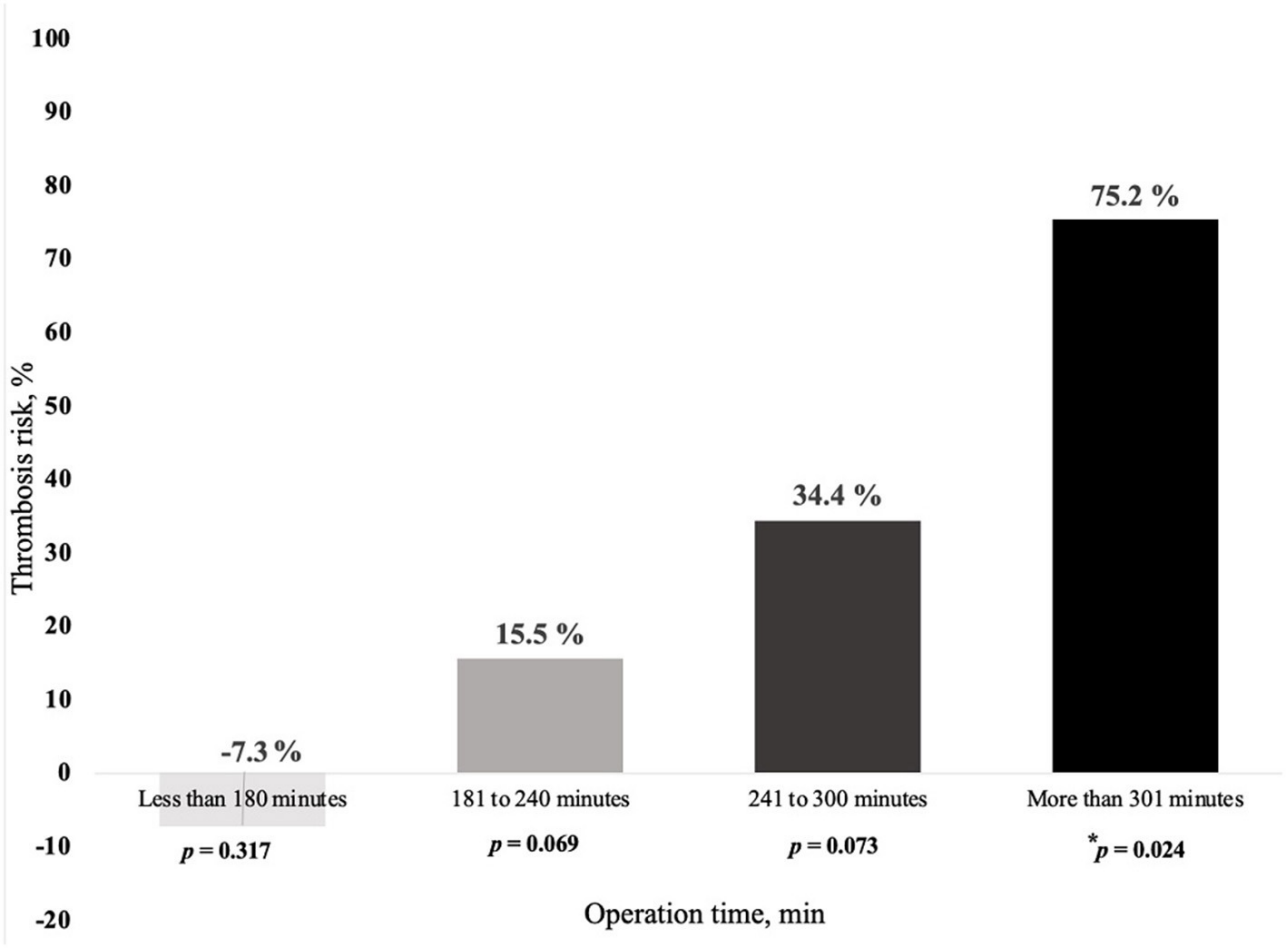
Relationship between risk of free flap thrombosis and duration of surgery in early surgery patients (ES group). In the ES group, the following subgroups were included as based on the duration of surgery (min, minutes), 19 patients <180 min, 48 patients from 181 to 249 min, 18 patients from 241 to 300 min and 18 patients > 300 min. Demographics, like age and sex did not differ significantly between the subgroups except for lower extremity surgery, which was performed less often in the subgroup with surgery time exceeding 300 min, respectively, *p* = 0.021. ^*^ denotes intergroup *p* = 0.024 between duration of surgery less and more than 300 min.

Duration of surgery appears to be a main predisposing factor for free flap thrombosis in the ES group. Prolonged duration of surgery demonstrated a tendency to promote development of postoperative thrombosis (OR = 1.007; *p* = 0.05), as depicted in Table 4. Moreover, we found that the risk of free flap thrombosis increased gradually with the duration of surgery as shown in Figure 1. Duration of surgery > 240 min increased the risk of free flap thrombosis 3.5 times, as determined with binary regression analysis (OR 3.5, CI 1.16–10.6; *p* = 0.026). In addition, linear regression analysis showed that surgery time from 240 up to 300 min increased the risk of free flap thrombosis by 34%.

**Figure 2:**
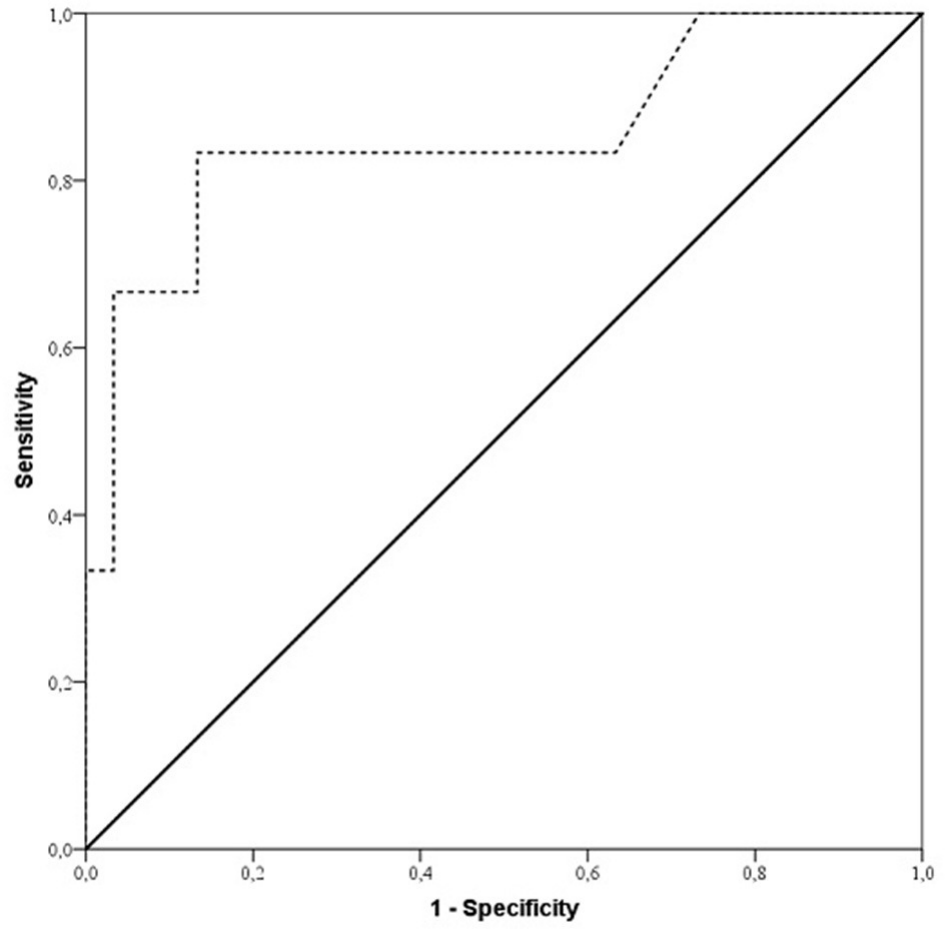
ROC curve showing sensitivity and specificity of duration of surgery for predicting free flap thrombosis risk in early surgery patients (ES group). Sensitivity and specificity were 93.3 and 86.7%, respectively (AUC 0.85; *p* = 0.007), for duration of surgery with a cut off value of 240 min of increased risk of free flap thrombosis in ES group patients.

According to ROC analysis (Figure 2), duration of surgery exceeding 240 min in ES group patients revealed a diagnosis of free flap thrombosis with a sensitivity of 93.3% and specificity of 86.7% and an AUC of 0.85; *p* = 0.007.

## Discussion

In microvascular surgery, a score, which identifies patients at increased risk of free flap thrombosis, is still lacking. The present prospective study of 103 patients with traumatic injuries, who underwent microvascular surgery, demonstrates that assessment of RTE data can be a useful tool for detection of hypercoagulability, although its prognostic value varies depending on the elapsed time after trauma. RTE might be convenient for predicting free flap thrombosis, particularly in patients who undergo elective surgery later than 30-days after a traumatic injury. The presence of hypercoagulability, which is often associated with thrombogenic comorbidities, increases the risk of free flap thrombosis as well. Although most patients in the ES group presented with posttraumatic hypercoagulability, the duration of surgery appeared to play an important role, independent of age, sex and localization of trauma and tissue defect.

Most investigators agree that traumatic injury promotes hypercoagulability. This occurs within only a few hours of the injurious event and leads to an elevated risk of thromboembolic complications. Muller et al. (31), analyzed RTE data from 886 trauma patients and observed that 10 % had hypercoagulability already during the first 24 h upon admission. Correspondingly, Selby and co-workers (35), examining 135 victims of major traumas, demonstrated an overall incidence of venous thromboembolism (VTE), including asymptomatic deep vein thrombosis in 59% of the patients. The authors also validated various coagulation and fibrinolysis markers for a potential role as predictors of VTE, but without pointing out any factor with such capability. Increased age was the only significant independent predictor of VTE.

During the recent few years, several studies have validated the ability of viscoelastic assays like RTE for monitoring of patients at increased risk of thrombophilia and thrombotic events (36–38). Thus, we chose RTE as the main diagnostic tool for assessing patients that potentially could be prone to free flap thrombosis. Of all the functionalities offered by this technology, the fibrinogen to platelet ratio (FPR) of RTE was considered to be the main index of hypercoagulability (32).

In the present study, free flap thrombosis developed in 15.5% of the patients regardless of time period after the traumatic event. Despite reports of progressive hypercoagulability and posttraumatic thrombotic complications following major surgery (39, 40), we miss data of hypercoagulability after trauma, that can predict the course of microvascular free flap thrombosis. We believe that coagulation activity escalates during the posttraumatic period. Based on our findings with RTE, 23% of all patients demonstrated hypercoagulability. Although incidence of FPR ≥ 42 in the ES group was significantly higher (44.4%) as compared with the LS group (11.9%), it did not correlate with the incidence of free flap thrombosis. Moreover, we showed that in patients with RTE data consistent with hypercoagulability, the incidence of free flap thrombosis was 25 vs. 12.7% in those without hypercoagulability, albeit the difference did not reach significance. A similar trend was also reported by Kolbenschlag and co-authors, who demonstrated RTE-based hypercoagulability in 36.5% of the patients who underwent microvascular surgery and an incidence of free flap thrombosis reaching 15.5% (34). The authors concluded that hypercoagulability detected by RTE at a FPR ratio > 43 identified patients likely to have thrombotic free flap complications. This corresponds to our findings. However, when hypercoagulability was associated with thrombogenic co-morbidities, free flap thrombosis rate was higher. In comparison with the study of Kolbenschlag et al., our ES patients were younger and had less thrombogenic co-morbidities. Therefore, we assume that hypercoagulability in association with thrombogenic comorbidities plays a more important role as compared with posttraumatic hypercoagulability.

According to Parker et al., FPR ≥ 42 might be a useful index for identifying patients at increased risk of contracting postoperative thrombosis after microvascular surgery (32). These authors included only patients with known head and neck tissue defects after oncological treatment. Moreover, they compared data from surgical patients with those of healthy volunteers assessing all thromboembolic complications. They demonstrated a predictive value of hypercoagulability, as assessed by RTE (with FRP ≥ 42) with a sensitivity of 89% and a specificity of 75% for thrombotic events. They found that nine (31%) of the patients had undergone some thromboembolic event and 8 of them had hypercoagulability with FPR ≥ 42. The authors noticed that patients with malignant diseases were at increased risk of venous thromboembolism *per se*.

In our cohort of 103 patients the main factors, revealing significant correlations with higher postoperative flap thrombosis rate, was the presence of thrombotic comorbidities in the LS group and the duration of surgery in the ES group. Previous investigators have demonstrated that smoking history (41), chronic obstructive pulmonary disease (42), diabetic complications (15), chronic cardiac failure (18) and peripheral vascular disease (6), all promote flap failure. We registered the history of previous thrombosis of any origin, such as ischemic disease, hypertension, obesity, neuroparesis, and diabetes as predisposing factors. It should be noted that LS group patients had significantly more frequent thrombotic comorbidities as compared with patients in the ES group. A possible explanation could be that younger people are more active and prone to traumas, whereas postponed reconstruction might occur more frequently in older ages, when health problems of various kinds have become manifest. We also observed, that LS patients were slightly older, 42.4 vs. 37.6 years. Regarding chronic osteomyelitis as a late posttraumatic complication, it was registered exclusively in 22 out of 67 patients (32.8%) of the LS group. We did not find any correlation between osteomyelitis and free flap thrombosis rate.

Whereas, most published studies have focused on thrombotic complications in oncological reconstruction surgery (6, 41, 43, 44) or a mixed population (42), few papers only have analyzed trauma cases (45). Moreover, only a few studies have assessed free flap thrombosis risk and the time elapsed between the traumatic event and microvascular reconstruction (14). To date, no one study has considered a possible influence of RTE-based hypercoagulability on flap thrombosis risk by comparing patients operated in an “early” posttraumatic period of up to 30-days after injury and a postponed reconstruction on an elective basis (later than 30 days).

Notably, Wikner and co-authors argued that RTE could be recommended only for assessment of anticoagulation therapy in free flap surgery patients (33). In our study, the surgeons ordinated enoxaparin 40 mg postoperatively as a standard thrombosis prophylaxis without individual risk assessment. Data regarding anticoagulation regimens still remain inconclusive. Clinical studies comparing various regimens have shown a reduction in the incidence of thrombotic complications, including anastomotic thrombosis and flap loss (43, 46). In general, anticoagulation therapy is administered, mostly because of difficulties in specifically detecting patients at increased thrombotic risks (34).

There are some limitations of our study. Although, all patients received standardized treatment, and were operated by experienced plastic surgeons routinely performing microvascular surgery, we consider technical surgical factors as the most actual limitations. Anyhow, we were not able to avoid the multifactorial nature of a thrombotic event and the human factor in surgery and postoperative care. In microvascular flap thrombosis, both the arterial and venous pools may play a role, which is further augmented by the genetic background of the patients (47, 48) or acquired thrombophilia. Therefore, we warrant an appropriate thrombotic risk assessment score for microvascular surgery patients. The CHAD2S2-VASc and the Caprini scores have been used to evaluate the risks of stroke in patients with atrial fibrillation and the risks of overall thromboembolic events in various patient populations. Nevertheless, we have no access to a method confidently estimating the risks of microvascular flap thrombosis (49–51). Moreover, we have not calculated the overall thrombosis risks preoperatively, which we denote as a weakness of the study. In addition, we did not address the possible influence of thrombosis promoting factors with regard to the type and severity of the primary injury in this patient population. Several investigators have analyzed the outcomes of free flap transfer surgery in certain body areas, such as head, neck and breast (52–55). Recently, Cho et al. demonstrated a positive predictive value of preoperative thrombocytosis for free flap thrombosis in acute trauma patients, but only in lower extremity reconstruction surgery (14). Finally, low incidence of RTE-detected hypercoagulability and free flap thrombosis rate in the LS group, and non-normally distributed data, revealed a rather wide confidence interval.

We were not able to show significant sensitivity and specificity of RTE - detected hypercoagulability and risk of free flap thrombosis in ES patients, although the patients often presented with posttraumatic hypercoagulability. Sample size was undersized as the statistical power was 69%, as tested with general linear model repeated measures test. However, assuming an incidence of free flap thrombosis of 15.5% in the whole population, and 33.3% in ES patients with RTE hypercoagulability, would reach a significance level of ≤ 0.05 at a power of 80% for the development of free flap thrombosis by increasing sample size in the ES group to 39 patients with hypercoagulability. So, admittedly the present study was not powered for this estimation.

## Conclusions

Thromboelastometry supports the detection of hypercoagulability and might have a predictive value for detection of free flap thrombosis in LS patients with thrombotic comorbidities. Although more patients were presented with posttraumatic hypercoagulability in the ES group, it is not predictive for free flap thrombosis. Prolonged surgery time (>240 min) should be considered as a major risk factor in these patients.

## Data Availability Statement

The datasets generated for this study are available on request to the corresponding author.

## Ethics Statement

The studies involving human participants were reviewed and approved by Riga Stradins University Ethics Commitee. The patients/participants provided their written informed consent to participate in this study.

## Author Contributions

BM, JS, and AO conceived the study. JS was responsible for data collection and informed the patients. IV, JS, and MM performed data analysis and JS made interpretation of data. JS, AO, and LB drafted and revised the manuscript. All authors read and approved the final manuscript, contributed substantially to the study design, analysis and interpretation of data, and drafting or revising the article.

## Conflict of Interest

The authors declare that the research was conducted in the absence of any commercial or financial relationships that could be construed as a potential conflict of interest.
